# Posterior Cingulate Cortex Network Predicts Alzheimer's Disease Progression

**DOI:** 10.3389/fnagi.2020.608667

**Published:** 2020-12-15

**Authors:** Pei-Lin Lee, Kun-Hsien Chou, Chih-Ping Chung, Tzu-Hsien Lai, Juan Helen Zhou, Pei-Ning Wang, Ching-Po Lin

**Affiliations:** ^1^Institute of Neuroscience, National Yang-Ming University, Taipei, Taiwan; ^2^Brain Research Center, National Yang-Ming University, Taipei, Taiwan; ^3^Department of Neurology, School of Medicine, National Yang-Ming University, Taipei, Taiwan; ^4^Department of Neurology, The Neurological Institute, Taipei Veterans General Hospital, Taipei, Taiwan; ^5^Department of Neurology, Far Eastern Memorial Hospital, New Taipei, Taiwan; ^6^Department of Medicine, Yong Loo Lin School of Medicine, National University of Singapore, Singapore, Singapore; ^7^Center for Cognitive Neuroscience, Neuroscience & Behavioral Disorders Program, Duke-National University of Singapore Medical School, Singapore, Singapore

**Keywords:** Alzheimer's disease, mild cognitive impairment, structural covariance network, synchronized degeneration network, hippocampus, posterior cingulate cortex

## Abstract

Alzheimer's disease (AD) is a progressive neurodegenerative disorder characterized by the accumulation of toxic misfolded proteins, which are believed to have propagated from disease-specific epicenters through their corresponding large-scale structural networks in the brain. Although previous cross-sectional studies have identified potential AD-associated epicenters and corresponding brain networks, it is unclear whether these networks are associated with disease progression. Hence, this study aims to identify the most vulnerable epicenters and corresponding large-scale structural networks involved in the early stages of AD and to evaluate its associations with multiple cognitive domains using longitudinal study design. Annual neuropsychological and MRI assessments were obtained from 23 patients with AD, 37 patients with amnestic mild cognitive impairment (MCI), and 33 healthy controls (HC) for 3 years. Candidate epicenters were identified as regions with faster decline rate in the gray matter volume (GMV) in patients with MCI who progressed to AD as compared to those regions in patients without progression. These epicenters were then further used as pre-defined regions of interest to map the synchronized degeneration network (SDN) in HCs. Spatial similarity, network preference and clinical association analyses were used to evaluate the specific roles of the identified SDNs. Our results demonstrated that the hippocampus and posterior cingulate cortex (PCC) were the most vulnerable AD-associated epicenters. The corresponding PCC-SDN showed significant spatial association with the patterns of GMV atrophy rate in each patient group and the overlap of these patterns was more evident in the advanced stages of the disease. Furthermore, individuals with a higher GMV atrophy rate of the PCC-SDN also showed faster decline in multiple cognitive domains. In conclusion, our findings suggest the PCC and hippocampus are two vulnerable regions involved early in AD pathophysiology. However, the PCC-SDN, but not hippocampus-SDN, was more closely associated with AD progression. These results may provide insight into the pathophysiology of AD from large-scale network perspective.

## Introduction

The human brain is traditionally considered to be a patchwork composed of neurons with specific functions and has been thoroughly dissected into histologically distinct regions based on functional organization or cellular cytoarchitecture. Advances in neuroimaging techniques have generated a novel view of the brain as a complex interconnected system that exerts its functions via both local and long-range connections (Biswal et al., 1995). Although the exact roles of these large-scale brain networks are not fully understood, disruptions of these networks have been demonstrated in various neurological diseases (Ahmed et al., 2016).

A pathological hallmark of neurodegenerative diseases is misfolded protein deposition in specific brain areas. In patients with Alzheimer's disease (AD), β-amyloid and tau proteins are widespread in many cortical regions and are correlated with clinical symptoms and cognitive functions (Braak and Braak, 1991). Recent studies further suggested that these misfolded proteins may be deposited in certain vulnerable anatomical regions early on, and spread along their corresponding large-scale networks in the brain as the disease progresses (Pievani et al., 2014; Franzmeier et al., 2020). According to this brain network degeneration hypothesis, the process may begin in epicenters of disease-specific networks, which are specific brain regions that are structurally and/or functionally vulnerable to the disease (Seeley et al., 2009). Misfolded proteins then spread along corresponding brain networks rather than by geographical proximity (Iba et al., 2013). Based on this hypothesis, several cross-sectional studies have identified AD epicenters as brain areas with maximal atrophy in patients with AD compared to healthy controls (HCs). These epicenters were then used as seeds to determine their corresponding structural and functional brain networks in HCs (Seeley et al., 2009; Dickerson et al., 2017). However, the epicenters identified using this approach may not be the earliest disease-involved regions, as AD pathology accumulates in the brain prior to the onset of clinical symptoms (Jack et al., 2010). In addition, it is also unclear whether these identified brain networks are associated with disease progression.

To identify AD-associated structural brain networks based on characteristics of disease progression, a 3-year-prospective study was conducted and the epicenters were posited as regions with greater annual atrophy rates in gray matter volume (GMV) in patients with mild cognitive impairment (MCI) who progressed to AD during the follow-up period as well as AD patients who were at an earlier stage. These regions were used as candidate epicenters to establish synchronized degeneration networks (SDNs) based on covariance patterns of annual GMV atrophy rates in HCs. This approach has been proposed as a surrogate marker for investigating longitudinal changes in large-scale structural networks (Alexander-Bloch et al., 2013). In contrast with the widely-used structural covariance network approach, which models the cross-sectional co-variance pattern of morphometric features across the study participants, the SDN approach uses longitudinal GMV atrophy rates as a coupling factor to construct the related structural network. Consequently, brain networks established using the SDN approach would more likely capture the progressive characteristic of neurodegenerative disease. We hypothesized that large-scale SDNs established with our identified epicenters could predict disease progression and provide further evidence supporting the network degeneration hypothesis for AD pathophysiology from a longitudinal perspective.

## Materials and Methods

### Participants

Patients with amnestic MCI, patients with AD dementia, and HCs were recruited for the study. During the 3-year follow up, patients with MCI who progressed to AD were classified as MCIp; those that remained stable were classified as MCIs. All patients were recruited from the memory clinic at Taipei Veterans General Hospital (TVGH), Taiwan. Before the study began, written informed consent was obtained from all participants and guardians for AD patients. This study was approved by the Local Ethics Committee of Human Research in TVGH (N0.97-04-1OA). Every subject was interviewed by the neurologist for history-taking and neuropsychological evaluation. Laboratory and MR examinations were used to exclude other major neurological diseases such as tumors, strokes, and severe white matter disease. None of the participants had a history of major head injury, brain tumor, stroke, epilepsy, alcoholism, major psychiatric illness, or other systemic diseases affecting cognitive function. HCs were volunteers with no neurological disease and no cognitive complaints.

### Clinical Assessments

The Mini-Mental State Examination (MMSE) was administered to assess global cognitive function (Folstein et al., 1975). To evaluate performance in different cognitive domains, the following cognitive tests were used:

- Verbal memory: the Chinese version of the Verbal Learning Test (CVVLT; nine items, four trials, and 10-min delayed recall) (Chang et al., 2010)
- Language: the categorical (animals) Verbal Fluency Test (VFT) and 30-item Boston Naming Test (BNT) (Cheung et al., 2004)
- Visuospatial function: the modified Rey-Osterrieth Complex Figure Test (CFT) (Boxer et al., 2003)
- Executive function: the modified Trail-Making Test, Part B (TMT-B) (Kramer et al., 2003)

The diagnosis of AD was based on the criteria from the National Institute of Neurological and Communicative Disorders and Stroke-Alzheimer's Disease and Related Disorders Association (NINCDS/ADRDA) (McKhann et al., 1984). All patients with AD had mild dementia with a baseline CDR score of 1 at the time of enrollment. All patients with amnestic MCI fulfilled the Petersen criteria (Petersen et al., 1999): (1) memory complaints, preferably corroborated by an informant; (2) objective memory impairment (verbal memory test, CVVLT ≤5, below 1.5 standard deviations of normal data) (Chang et al., 2010); (3) normal general cognitive function (MMSE ≥24); (4) intact daily living activities; and (5) dementia criteria not met. As defined for amnestic MCI, only patients with isolated memory impairments and without neuropsychological evidence of dysfunction in other cognitive domains were recruited. All participants were scheduled to receive clinical and imaging tests annually for 3 years. Only subjects that received at least two MRI examinations (17 participants with 2 consecutive scans and 76 participants with 3 consecutive scans) were included in subsequent analyses.

### Image Acquisition

The MRI scans were acquired using an eight-channel phased-array head coil on the identical 1.5 T Excite-II MRI scanner (General Electric Healthcare, Milwaukee, Wisconsin, USA) at TVGH. Foam pads were used to minimize head movement during image acquisition. T1 weighted anatomical images were acquired using a 3-dimensional fluid-attenuated inversion-recovery fast spoiled gradient recalled echo sequence with the following imaging parameters: repetition time/echo time/inversion time = 8.548/1.836/400 ms; flip angle = 15 degrees; number of excitations = 1; matrix size = 256 × 256 × 124 without inter-slice gap and interpolation; and voxel size = 1.02 × 1.02 × 1.50 mm^3^. Each individual brain scan was manually inspected for image artifacts and gross anatomical abnormalities by an experienced radiologist before the morphometry analysis. No participant was excluded for brain abnormalities. Before subsequent image processing, we reoriented all images to have an approximate point of origin at the anterior commissure.

### Image Analysis

**Figure 1:**
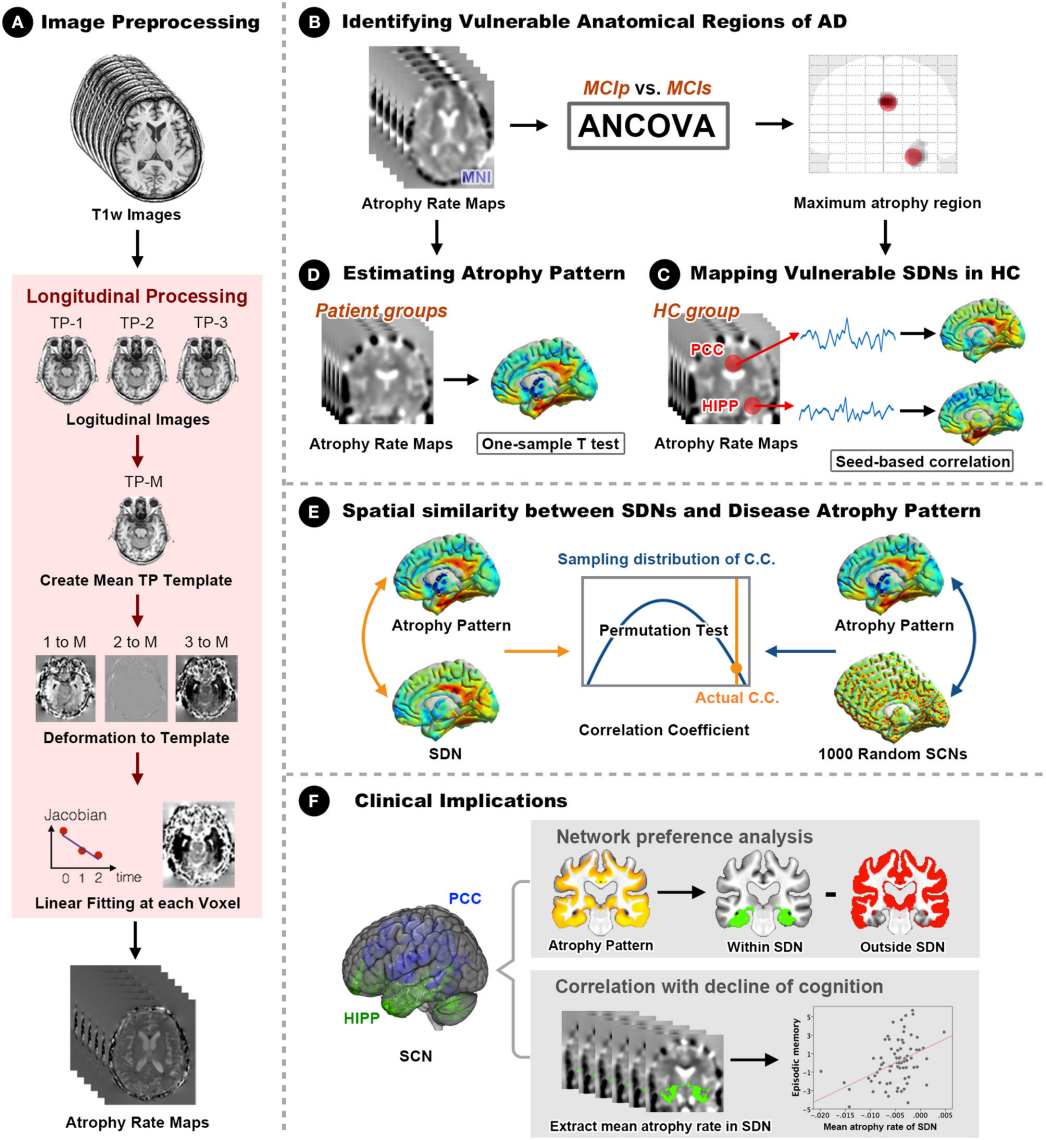
Framework of the study design and analyses. **(A)** The optimized longitudinal anatomical image preprocessing pipeline was used to generate individual annual changing rate maps in Montreal Neurological Institute (MNI) standard space. **(B)** Vulnerable regions (hippocampus and PCC) were identified by comparing MNI space annual changing rate maps between MCIp and MCIs groups. **(C)** Seed-based correlation analyses were conducted to identify corresponding large-scale synchronized degeneration networks (SDNs) in the HC group. **(D)** A one-sample *t*-test was performed for each patient group to map group-specific degenerative patterns (different than zero) over time. **(E)** Permutation test was conducted to assess the spatial similarity between SDNs and group-specific degenerative patterns by comparing the strength of actual correlations with the distribution from randomly generated SDNs. **(F)** Network preference analysis and Spearman correlation analysis were conducted to investigate clinical implications. AD, Alzheimer's disease; ANCOVA, analysis of covariance; C.C., correlation coefficient; HC, healthy control; HIPP, hippocampus; MCIp, mild cognitive impairment with progression to AD; MCIs, mild cognitive impairment stable without progression to AD; PCC, posterior cingulate cortex; TP, time point; SDN, synchronized degeneration network.

A general overview of the analytical framework is illustrated in Figure 1. Details of the analytic pipeline are summarized below.

#### Estimating Individual Voxel-Wise Anatomical Changing Rate Map

To estimate voxel-wise anatomical brain changes over time and enable subsequent statistical analyses independent of the number of time points, a two-stage tensor-based morphometry approach was applied using Statistical Parametric Mapping software (SPM12 version 7487, Wellcome Institute of Neurology, University College London, UK, http://www.fil.ion.ucl.ac.uk/spm/) with default settings and in-house MATLAB codes (R2018a, Mathworks, Natick, MA). In the first stage, all available native space T1w scans for each individual were warped longitudinally to their corresponding midpoint average image using an inverse-consistent non-linear registration approach available in the “Serial Longitudinal Registration” module of SPM12 (Ashburner and Ridgway, 2012). Experiment times for each scan were entered into the registration algorithm, generating Jacobian determinant maps of each time point and the corresponding midpoint average images for each individual. All midpoint average anatomical images were subsequently segmented into three distinct tissue types (gray matter, white matter, and cerebrospinal fluid) using a unified segmentation approach (Ashburner and Friston, 2005). The resulting gray and white matter tissue segments were used to construct group-specific tissue templates and estimate deformation fields using a fast diffeomorphic image registration algorithm (Ashburner, 2007). This procedure enabled the transformation of individual Jacobian determinant maps into the standard Montreal Neurological Institute (MNI) space. Subsequently, to estimate voxel-wise changing rate maps, a linear regression model was applied to the MNI-space Jacobian determinant maps of each time point for each individual participant. The estimated slope of the regression model presented the changing rate of the brain across multiple time points. The resulting changing rate maps were then further smoothed using an isotropic 8 mm full-width at half-maximum Gaussian kernel. These preprocessed data encoded the relative speed of brain expansion or contraction per individual, and were used for subsequent voxel- and network-level analyses. To exclude partial volume effects of borders between different tissue types, individual unmodulated gray matter segments of corresponding midpoint average images were averaged and set at a threshold (0.2 intensity) to create explicit masks. Individual native space baseline T1w scans were used to estimate total intracranial volume.

#### Voxel-Wise Statistical Analyses of Changing Rate Maps

The GLM Flex toolbox (http://mrtools.mgh.harvard.edu/index.php?title=GLM_Flex) with appropriate statistical models was used for the following voxel-wise statistical analyses. A single-factor-four-level (HC, MCIp, MCIs, and AD) analysis of covariance with age, sex, educational years, and total intracranial volume as nuisance covariates was used to identify between-group differences in GMV changing rates of local brain areas. A separate one-sample *t*-test was performed for each study group to map group-specific degenerative patterns (different from zero) over time. Voxel-wise statistical results were set at a voxel-level uncorrected *p* < 0.005 and extent threshold of family wise error (FWE) corrected *p* < 0.05 (cluster extent = 513 voxels) using the updated “3dFWHMx” and “3dClustSim” programs implemented in the Analysis of Functional Neuroimages software (AFNI, version 19.3.17). For transparency and reusability of statistical results, all unthresholded statistical maps of direct group comparisons and group-specific degenerative patterns can be downloaded from the NeuroVault website (https://neurovault.org/collections/3273/).

#### Disease-Specific Epicenter Identification and Synchronized Structural Degeneration Network Analysis

Disease-specific epicenters for synchronized SDN analysis were identified by placing 6-mm-radius spheres at the most-significant voxel from the above direct-group voxel-wise changing rate analysis (MCIp vs. MCIs). Brain regions with significantly higher GMV changing rates in MCIp were defined as early AD-associated epicenters and further selected as seed regions-of-interest (ROIs) for mapping large-scale SDNs in HCs. In accordance with previous longitudinal studies (Alexander-Bloch et al., 2013), we extracted mean changing rate values of seed ROIs (hippocampus and the posterior cingulate cortex [PCC]) and then entered these values into respective general linear models to identify possible coupling patterns between seed ROIs and voxels across the rest of the brain in HCs. The same nuisance covariate settings and statistical criteria were used for SDN analyses. Unthresholded statistical maps of these SDNs are also available at the NeuroVault website (https://neurovault.org/collections/3273/).

#### Spatial Similarity and Network Preference Analyses

To investigate whether AD-specific SDNs predicted the longitudinal GMV atrophy rate in each disease group, a voxel-wise spatial cross-correlation approach was used to assess similarities between spatial distributions of unthresholded group-specific degenerative maps and SDN maps (Douaud et al., 2014). Using non-parametric permutation tests to assess the statistical significance of observed spatial relationships, 1,000 random Gaussian noise maps were generated and smoothed with corresponding estimated smoothness from SDN maps. We then calculated 1,000 spatial cross-correlations between simulated SDNs and group-specific degenerative maps, and compared the strength of observed correlations with the empirically generated null distribution. To test whether AD-specific SDNs exhibited different vulnerability levels in each disease group, network preference analysis was conducted (Seeley et al., 2009). First, binarized network-level ROIs were generated from previous SDN analysis with statistical thresholding (cluster-level FWE corrected *p* < 0.05). We then calculated the goodness-of-fit (GOF) score between binarized network ROIs and group-specific degenerative patterns (from previous one-sample *t*-tests of voxel-wise changing rate map analysis). The GOF score reflected how well SDNs fit each group-specific degenerative pattern, and was defined by the difference between the mean *z*-value within and outside the binarized network ROIs. Furthermore, to confirm the stability and reliability of the results of the network preference analysis, we performed an additional GOF analysis using binarized network-level ROIs with fixed network size. More specifically, we first ranked the whole brain voxels from highest to lowest according to the corresponding voxel-wise *z*-value of PCC- and hippocampus-epicentered network analyses. After voxel ranking procedure, 10 binarized network ROIs with different network sizes from the top 1 to 10 percent of all brain voxels with 1 percent intervals were generated for the PCC- and hippocampus-epicentered SDNs, respectively. This procedure provides a more identical network size for both AD-specific SDNs to be used in the GOF-based network preference analysis. The GOFs scores were then calculated using the same approach which was mentioned above.

#### Relationship Between Cognitive Decline and the Mean Changing Rate of AD-Specific Synchronized Structural Degeneration Networks

To investigate the relationships between AD-specific SDNs and cognitive decline, mean GMV changing rates of AD-specific SDNs were extracted, averaged, and entered into MATLAB software to perform partial Spearman's rank order correlation analysis with the changing rate of neuropsychological test scores. Participants' age, sex, education years, and total intracranial volume were included as nuisance variables. A Bonferroni correction was applied to correct for multiple comparisons for correlation analyses, excluding the MMSE which was considered a separate test representing global cognition. The threshold for statistical significance was set at corrected *p* < 0.05.

### Statistical Analyses of Demographic, Clinical Characteristics, and Global Tissue Volume at Baseline

The statistical analyses of non-voxel-wise data were performed with IBM SPSS Statistics Version 25 (Armonk, NY). We used the Shapiro-Wilk normality test to check that each variable was normally distributed. The Chi-square test was used to examine categorical data. The Analysis of Variance and Kruskal-Wallis rank sum tests were used to identify differences in continuous variables after considering distributional assumptions. Two-tailed *p* < 0.05 were considered statistically significant.

## Results

### Patients' Characteristics and Clinical Data

In total, 23 patients with AD, 37 patients with MCI, and 33 HCs were included at baseline. During the 3-year follow-up, 12 of the patients with MCI progressed to AD (MCIp); the remaining 25 patients remained stable (MCIs). Patient demographics and baseline cognitive function test results are listed in Table 1. Age and sex were similar among study groups. Differences in education years were noted. *Post-hoc* analysis revealed greater education years in HCs than in MCIp (*p* = 0.011) and AD patients (*p* = 0.036). Significant differences were observed in the baseline cognitive function test results between study groups with the exception of the complex figure test copy section (CFT copy). In the majority of tests, HCs performed better than MCIs, followed by MCIp and AD.

**Table 1:** Demographics and baseline clinical characteristics.

	**HC**	**MCIs**	**MCIp**	**AD**	***p*-value**	***Post-hoc* comparisons**
Number	33	25	12	23		
Female [*n*, (%)]	18 (54.5%)	16 (64.0%)	6 (50.0%)	12 (52.2%)	0.804^a^	
Age (years)	74.9 (5.46)	77.1 (6.08)	77.1 (6.24)	78.3 (5.79)	0.066^b^	
Education (years)	13.1 (3.21)	12.4 (3.53)	9.58 (4.48)	10.3 (4.87)	0.035^b^	HC>MCIp/AD
MMSE	28.5 (1.42)	27.2 (1.87)	24.7 (2.50)	20.7 (2.86)	<0.001^b^	HC>MCIs>MCIp>AD
CVVLT	7.79 (1.29)	5.04 (1.43)	3.50 (2.81)	1.04 (1.40)	<0.001^c^	HC>MCIs/MCIp>AD
CFT copy	15.7 (1.57)	15.4 (1.71)	14.8 (2.01)	15.0 (1.72)	0.352^b^	
CFT recall	11.5 (3.28)	6.96 (3.43)	4.58 (4.19)	0.83 (1.85)	<0.001^b^	HC>MCIs/MCIp>AD
VFT	16.8 (3.88)	12.7 (3.08)	13.6 (3.85)	12.3 (4.08)	<0.001^b^	HC>MCIs/MCIp/AD
BNT	28.2 (2.32)	26.6 (2.23)	24.5 (3.50)	23.5 (3.36)	<0.001^b^	HC>MCIs/MCIp/AD; MCIs>AD
TMT-B lines	13.3 (2.28)	11.4 (3.76)	11.2 (3.97)	8.59 (5.03)	<0.001^b^	HC>MCIs/MCIp/AD; MCIs>AD
GMV (cm^3^)	592 (57.1)	578 (56.9)	569 (51.5)	544 (47.0)	0.015^c^	HC/MCIs>AD
WMV (cm^3^)	398 (50.8)	399 (47.5)	385 (46.0)	375 (33.9)	0.230^c^	
CSFV (cm^3^)	471 (92.7)	504 (80.4)	506 (88.9)	499 (87.5)	0.073^c^	
TIV (cm^3^)	1,460 (146)	1,481 (118)	1,461 (152)	1,451 (131)	0.895^c^	

a *Chi-square test was used for group comparison in categorical variables*.

b *Kruskal-Wallis rank sum test was used for comparing of group differences in continuous variables with non-normal distributions*.

c *Analysis of variance test was used for comparing group differences in continuous variables with a normal distribution*.

*AD, Alzheimer's disease; BNT, Boston Naming Test; CFT, Complex Figure Test; CSFV, Cerebrospinal fluid volume; CVVLT, Chinese version of the Verbal Learning Test; GMV, gray matter volume; HC, healthy controls; MCIp, mild cognitive impairment-progression; MCIs, mild cognitive impairment-stable; MMSE, Mini-Mental Screening Examination; TIV, total intracranial volume; TMT-B lines, Trail-making Test Part B lines completed in 120 s; VFT, Verbal Fluency Test; WMV, white matter volume*.

### Epicenter Identification and Group Differences in Annual GMV Atrophy Rate

**Figure 2:**
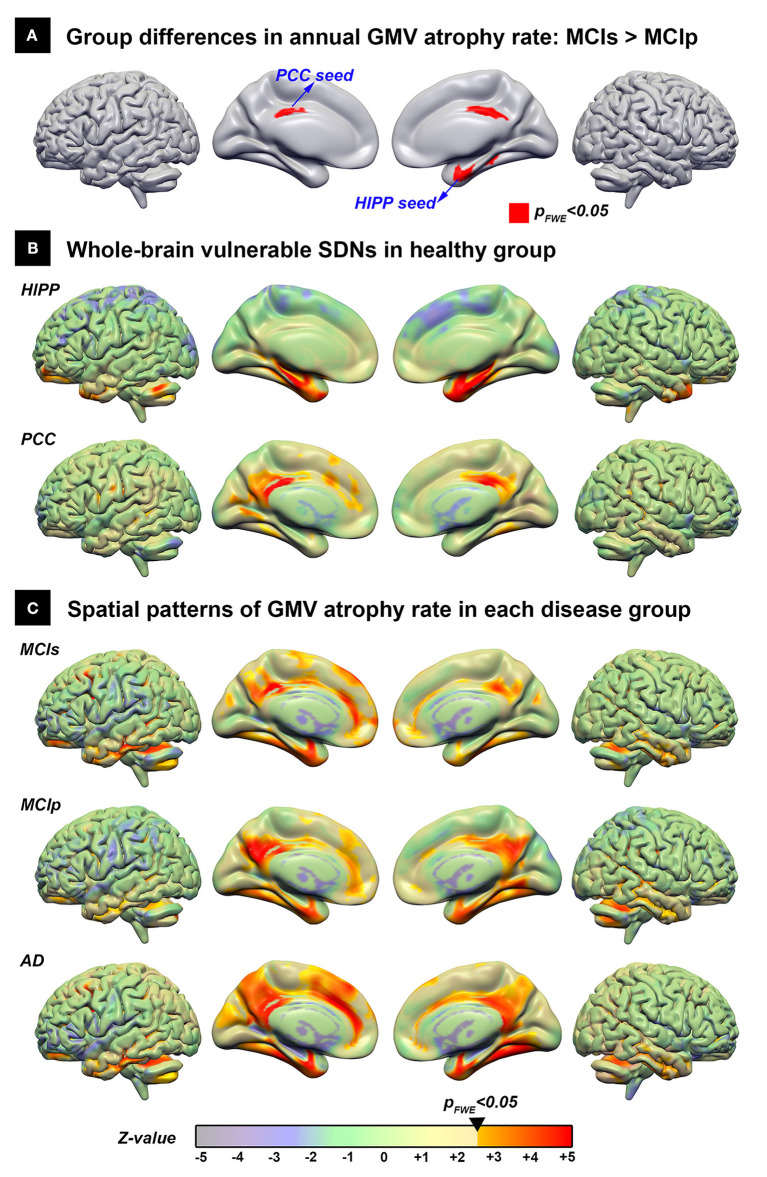
The spatial distribution of the vulnerable SDNs and GMV atrophy rate patterns in disease groups. **(A)** Direct group comparison of the annual gray matter atrophy rate between MCIs and MCIp groups to identify early AD-associated epicenters. **(B)** Whole brain vulnerable SDNs illustrated in the HC group by seed-based correlation analyses in the epicenters on **(A)**. **(C)** Group-specific spatial patterns in each patient group based on one-sample *t*-tests. The transparent colors indicate the *z*-value of statistical results without a significant threshold; the solid colors show the significant regions. AD, Alzheimer's disease; HC, healthy control; HIPP, hippocampus; GMV, gray matter volume; MCIp, mild cognitive impairment with progression to AD; MCIs, mild cognitive impairment stable without progression to AD; PCC, posterior cingulate cortex; SDN, synchronized degeneration network.

Using the direct group comparison of voxel-wise annual GMV atrophy rate maps between patients with MCIp and MCIs, the hippocampus and PCC were identified as the early AD-associated disease epicenters (Figure 2A). Additionally, all possible group differences in the regional GMV atrophy rate are illustrated in Supplementary Figure 1; detailed anatomical locations are listed in Supplementary Table 1. Overall, the AD group had the fastest atrophy rate, followed by MCIp, MCIs, and HC groups. More specifically, compared to HCs, patients with AD had faster atrophy rates in the hippocampus, temporal pole, frontal lobe, cingulate gyrus, and cuneus/precuneus. No brain areas exhibited a decreased annual GMV atrophy rate when comparing disease groups and HCs.

### Spatial Distribution of Vulnerable SDNs

The spatial distribution of large-scale hippocampus- and PCC-epicenter SDNs are illustrated in Figure 2B and the detailed anatomical locations of the corresponding SDNs are listed in Supplementary Table 2. The hippocampus-epicentered SDN involved brain areas surrounding the hippocampus (parahippocampus, entorhinal cortex, temporal pole, and temporal fusiform cortex), frontal poles, and the cerebellum. On the other hand, the PCC-epicentered SDN included more widespread brain areas, including the cingulate, frontal lobe, temporal lobe, insula, and cerebellum.

### Spatial Similarity Between Vulnerable SDNs and GMV Atrophy Rate Patterns in Disease Groups

The voxel-wise spatial patterns of GMV atrophy rates for each disease group are illustrated in Figure 2C. Significant spatial correlation between atrophy patterns and SDNs were noted for PCC-epicentered SDN (MCIs: *r* = 0.571, *p* < 0.001; MCIp: *r* = 0.639, *p* < 0.001; AD: *r* = 0.570, *p* < 0.001) and hippocampus-epicentered SDN (MCIs: *r* = 0.285, *p* < 0.001; MCIp: *r* = 0.415, *p* < 0.001), with the exception of the hippocampus-epicentered SDN in the AD group (*r* = 0.1, *p* > 0.99).

### Network Preference Analysis Revealed the Specific Role of Each SDN

**Figure 3:**
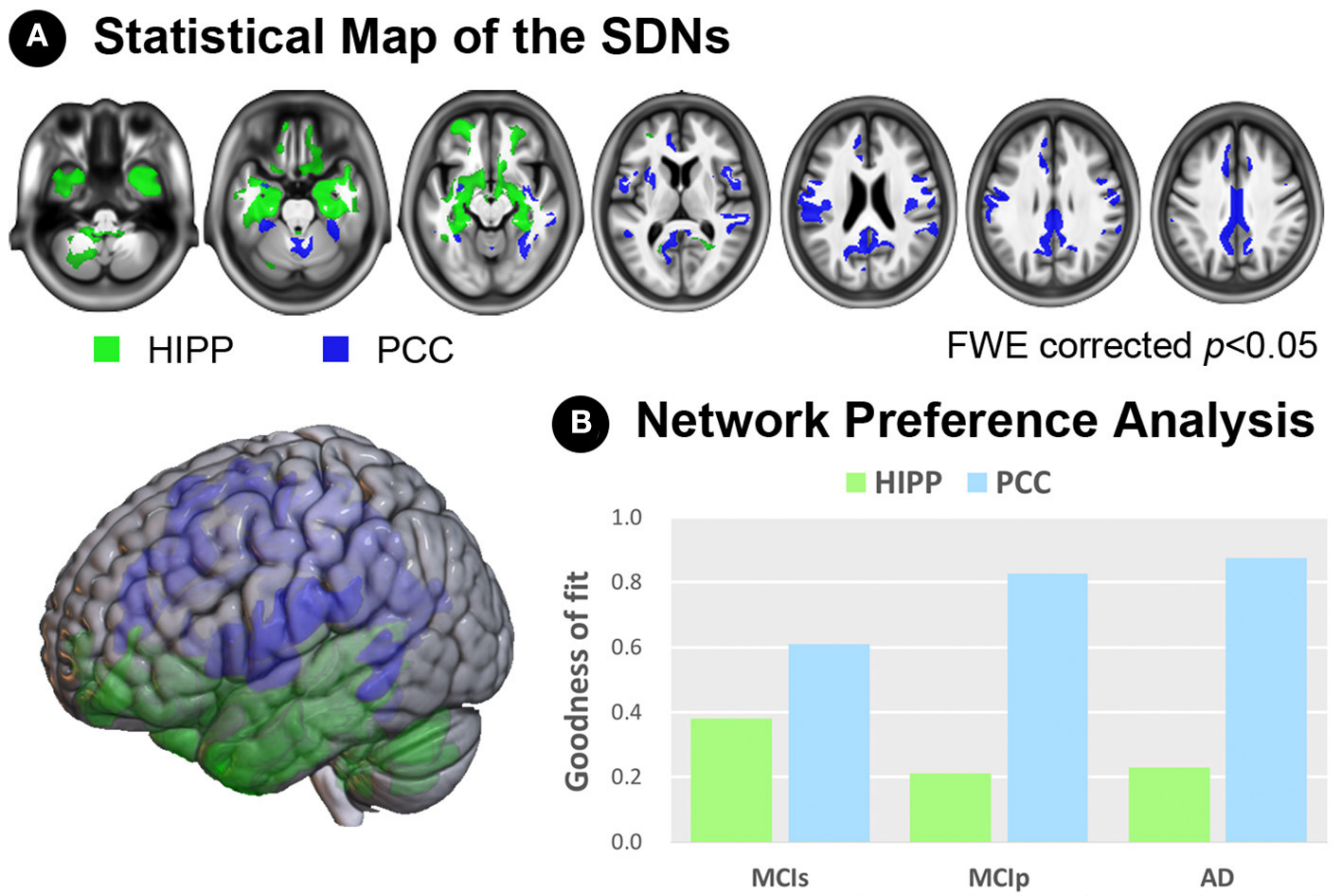
Network preference analysis. **(A)** Statistical maps of the hippocampus- and PCC-SDNs. **(B)** Preference determined by goodness-of-fit showed a stronger association of the PCC-SDN than that of the hippocampus-SDN, especially in the MCIp and AD groups. AD, Alzheimer's disease; FWE, family-wise error; HIPP, hippocampus; MCIp, mild cognitive impairment with progression to AD; MCIs, mild cognitive impairment stable without progression to AD; PCC, posterior cingulate cortex; SDN, synchronized degeneration network.

To examine network preferences across different disease stages, we investigated the fitness between hippocampus/PCC-epicentered SDNs and whole-brain atrophy rate patterns of all disease groups (Figure 3). We first generated binarized masks of hippocampus- and PCC-epicentered SDNs (FWE-corrected *p* < 0.05, Figure 3A), and calculated the GOF according to the different disease stages (Figure 3B). A higher GOF represented more similarity between the SDN and disease atrophy pattern. For the PCC-epicentered SDN, overlaps were more evident with more advanced disease stages (GOF scores in MCIs = 0.610; MCIp = 0.827; AD = 0.874). This trend was not observed for the hippocampus-epicentered SDN (GOF scores in MCIs = 0.380; MCIp = 0.213; AD = 0.230). Furthermore, the additional GOF-based network preference analysis, which uses a different degree of fixed size approach to determine the network ROIs, also demonstrated the same relationship between SDN and disease atrophy pattern across different disease stages (Supplementary Figure 2).

### Correlations Between the GMV Atrophy Rate of SDNs and Cognitive Decline

To test whether large-scale SDNs were associated with progressive cognitive decline, we performed exploratory correlation analyses between mean annual GMV atrophy rates of each SDN and deterioration slopes of neuropsychological test scores (Table 2). Significant correlations between PCC-epicentered SDN, but not hippocampus-epicentered SDN, and cognitive decline were observed in most domains (including MMSE, CVVLT, CFT recall, VFT, and BNT).

**Table 2:** Correlations between the mean annual gray matter volume atrophy rate and slopes of neuropsychological test scores.

	**Spearman rank order test correlation coefficients**			
	**Hippocampus-SDN**		**PCC-SDN**	
	**rho**	***p*-value**	**rho**	***p*-value**
MMSE	0.001	0.994	**0.245**	0.035^*^
CVVLT	0.093	0.432	**0.400**	**<0.001^†^**
CFT copy	−0.030	0.799	0.052	0.659
CFT recall	0.089	0.455	**0.336**	**0.004^†^**
VFT	0.150	0.202	**0.429**	** <0.001^†^**
BNT	0.298	0.010	**0.304**	**0.008^†^**
Trail B line	0.052	0.668	0.215	0.076

* *p < 0.05*.

† *p < 0.008 (statistically significant correlation after Bonferroni correction)*.

*BNT, Boston Naming Test; CFT, Complex Figure Test; CVVLT, Chinese version of the Verbal Learning Test; MMSE, Mini-Mental Screening Examination; PCC, posterior cingulate cortex; SDN, synchronized degeneration network; TMT-B lines, Trail-making Test Part B lines completed in 120 s; VFT, Verbal Fluency Test*.

*Significant values are bolded*.

## Discussion

The results from this 3-year longitudinal study support the network degeneration hypothesis of AD. Our results indicated that the PCC and hippocampus were the two most vulnerable regions involved in the early-stage of AD. Spatial correlation analysis further demonstrated PCC- and hippocampus-epicentered SDNs in HCs strongly associated with the GMV atrophy patterns of disease groups. However, only the PCC-epicentered SDN was associated with disease severity, and its GMV atrophy rate predicted cognitive decline in multiple domains. These findings collectively indicate the distinct roles of PCC- and hippocampus-epicentered SDNs in the pathophysiology of AD.

The hippocampus, which plays an important role in declarative memory, is the anatomical signature of AD (Schröder and Pantel, 2016). Hippocampal atrophy, and more specifically, its atrophy rate, may be potential biomarkers to predict the conversion from MCI to AD (Henneman et al., 2009). Our voxel-wise atrophy rate analyses supported the regional role of the hippocampus in AD progression. In addition to its regional significance, the hippocampus has also been shown to be an important node in several large-scale brain networks and has been implicated as part of the subsystem of the default mode network (DMN) (Andrews-Hanna et al., 2010). Decreased integrity of hippocampus-associated functional and structural networks has also been reported (Zhou et al., 2008; O'Callaghan et al., 2019). In this study, we used the coupling atrophy rate as a surrogate image marker for longitudinal mapping of potential large-scale brain SDNs. We identified the parahippocampus, temporal pole, temporal fusiform cortex, frontal poles, and cerebellum within a single hippocampus-epicentered SDN. Although network mapping approaches vary among studies, the spatial distribution of identified hippocampus-epicentered SDNs is highly accordant with previous studies (Bai et al., 2009; Zhu et al., 2018). Close connections between the hippocampus and nearby regions, collectively referred to as the medial temporal lobe, have been reported in various histopathological and neuroimaging findings of AD (Braak and Braak, 1985, 1991). Beyond the limbic system, considerable evidence indicates that the hippocampus and prefrontal cortex become coupled via oscillatory synchrony reflecting bidirectional information flow (Battaglia et al., 2011) and may play an important role in memory and learning (Eichenbaum, 2017). Taken together, these regional and network-level findings underscore the importance of the hippocampus and its corresponding functionally/structurally connected areas in AD pathophysiology.

The PCC is an area in the brain with higher metabolic activity and dense anatomical and functional connections to many other brain regions. PCC hypometabolism, volume atrophy, and connectivity corruption have been reported in patients with AD (Leech and Sharp, 2014). Longitudinal follow-up in patients with MCI revealed that changes in PCC connectivity over time were correlated with declines in MMSE and other cognitive test scores (Wang et al., 2012). From a global network perspective, the PCC is considered to be a central hub of the DMN and is inter-connected with several large-scale brain networks (Raichle et al., 2001). Based on its regional and global characteristics, previous studies have indicated that the PCC may be involved in multiple cognitive functions including autobiographical/episodic memory retrieval, attention, salience, attention, and emotion (Leech and Sharp, 2014). These domains of cognitive function also changed during AD progression (Mortamais et al., 2017). Among these multiple PCC-connected large-scale brain networks, DMN is the first and most consistently reported network to be involved in AD (Badhwar et al., 2017). DMN failure begins early in the course of AD, even prior to measurable amyloid accumulation (Jones et al., 2016). Furthermore, using various network mapping approaches, including intrinsic functional connectivity and the cross-sectional structural covariance method, previous studies have reported that the integrity of the PCC-epicentered DMN may be associated with the clinical severity and progression of AD (Zhang et al., 2010), suggesting that the PCC and its corresponding brain networks have important roles in AD progression.

In our study, the PCC-epicentered SDN involved widespread frontal, temporal, insular, and cerebellar areas. Most of these areas overlap with the classical DMN that includes the precuneus, medial and lateral parietal, medial prefrontal, and medial and lateral temporal cortices (Raichle, 2015). The insular cortex is a notable exception, as it is typically not included in the DMN. The insular cortex is a core limbic area, historically and phylogenetically associated with emotion, and may underpin the behavioral and emotional symptoms in AD (Spalletta et al., 2010). The insular and anterior cingulate cortices are key hubs of the salience network that is also involved in AD and MCI. The insula may play a role in connecting the salience network and DMN, switching from externally-oriented to internally-oriented mental status (Sridharan et al., 2008). Functional and structural disruptions to the switching mechanism occur with disease progression in patients with AD (Xie et al., 2012; Liu et al., 2018). On the other hand, the cerebellum was shown to be involved in the PCC-epicentered SDN. Although traditionally considered to be involved in motor coordination, recent studies have further suggested that the cerebellum may be involved in multiple domains of cognitive function based on its complex spatial connectivity profile with large-scale cortical brain networks (King et al., 2019). Beyond its spatial characteristics, recent intrinsic functional connectivity studies have further suggested that the cerebellum may engage in a domain-general function in the adaptive control of the cortical process which may impaired in the progression of AD (Bai et al., 2011; Zheng et al., 2017; Marek et al., 2018). Taken together, these findings suggest the potential importance of the cerebellum in the pathogenesis of AD.

The findings of our study demonstrated that compared to the hippocampus-epicentered SDN, the PCC-epicentered SDN atrophy rate was more strongly correlated with deterioration slopes of cognitive tests in multiple domains. Moreover, the PCC-epicentered SDN predicted AD progression better than did the hippocampus SDN. One possible explanation is that the hippocampus and the surrounding entorhinal cortex are involved earliest in the course of AD (Braak and Braak, 1991), which might suggest that further atrophy rate in the hippocampus SDN is not as relevant. In addition, compared to the hippocampus, the PCC may be an integrative hub which mediates information flow across whole-brain networks (Leech and Sharp, 2014). Although the PCC and hippocampus are both components of the DMN, and considering the different functional roles in the DMN (central vs. peripheral), we propose that deficits in the PCC-epicentered network may better represent overall AD progression in terms of structural changes and cognitive decline in multiple domains. In addition, the fact that we did not observe any correlation between hippocampus-epicentered network and cognitive decline might be due to the small sample size in the current study. Future studies with a larger sample size will be needed to confirm the potential role of core PCC region and related connected brain areas and to determine the exact mechanism of the involvement of this region in the pathophysiology of AD.

To the best of our knowledge, this study is the first to investigate the associations between structural network changes, brain volume atrophy, and cognitive decline using an SDN approach from a longitudinal perspective. One strength of our study was its longitudinal follow-up design, which enabled us to identify AD-related epicenters involved early in the course of AD. Additionally, we demonstrated a relationship between large-scale structural brain networks and AD progression. However, our results should be interpreted with caution; first, due to the longitudinal design, the dropout rate was high, limiting the generalizability of our results to large disease populations. However, our exploratory findings may guide future studies with larger samples. Second, AD and MCI diagnoses were made according to characteristic clinical presentation and neuropsychological performance. Although these criteria are widely accepted for both clinical and research purposes, potential bias may exist due to a lack of amyloid and tau biomarkers. Third, we defined the structural networks based on brain regions with maximal changes during the conversion from MCI to AD; earlier changes occurring during the progression from HC to MCI might have been overlooked.

In conclusion, the PCC and hippocampus are two vulnerable regions involved early in AD pathophysiology. Notably, the PCC-epicentered, but not hippocampus-epicentered, network predicts AD progression, including brain atrophy and cognitive decline. Our results support the network degeneration hypothesis of AD and suggest that PCC large-scale SDNs may be used as potential markers for disease progression. Further, the results provide insight regarding the mechanisms of network pathology in AD.

## Data Availability Statement

Unthresholded statistical maps of all voxel-wise analyses are available to download from the NeuroVault repository (https://neurovault.org/collections/3273/). Requests to access the datasets should be directed to Ching-Po Lin, cplin@ym.edu.tw.

## Ethics Statement

The studies involving human participants were reviewed and approved by Local Ethics Committee of Human Research in Taipei Veterans General Hospital (N0.97-04-1OA). The patients/participants provided their written informed consent to participate in this study.

## Author Contributions

C-PL and P-NW were responsible for study concept and design. P-LL and K-HC were responsible for data acquisition and analyses. P-LL, K-HC, C-PC, T-HL, and JZ were responsible for drafting the manuscript, tables, and figures. All authors contributed to the article and approved the submitted version.

## Conflict of Interest

The authors declare that the research was conducted in the absence of any commercial or financial relationships that could be construed as a potential conflict of interest.

## References

[B1] Ahmed R. M. Devenney E. M. Irish M. Ittner A. Naismith S. Ittner L. M.. (2016). Neuronal network disintegration: common pathways linking neurodegenerative diseases. J. Neurol. Neurosurg. Psychiatr. 87, 1234–1241. 10.1136/jnnp-2014-308350 27172939 PMC5099318

[B2] Alexander-Bloch A. Raznahan A. Bullmore E. Giedd J. (2013). The convergence of maturational change and structural covariance in human cortical networks. J. Neurosci. 33, 2889–2899. 10.1523/JNEUROSCI.3554-12.2013 23407947 PMC3711653

[B3] Andrews-Hanna J. R. Reidler J. S. Sepulcre J. Poulin R. Buckner R. L. (2010). Functional-anatomic fractionation of the Brain's default network. Neuron 65, 550–562. 10.1016/j.neuron.2010.02.005 20188659 PMC2848443

[B4] Ashburner J. (2007). A fast diffeomorphic image registration algorithm. Neuroimage 38, 95–113. 10.1016/j.neuroimage.2007.07.007 17761438

[B5] Ashburner J. Friston K. J. (2005). Unified segmentation. Neuroimage 26, 839–851. 10.1016/j.neuroimage.2005.02.018 15955494

[B6] Ashburner J. Ridgway G. R. (2012). Symmetric diffeomorphic modeling of longitudinal structural MRI. Front. Neurosci. 6:197. 10.3389/fnins.2012.00197 23386806 PMC3564017

[B7] Badhwar A. Tam A. Dansereau C. Orban P. Hoffstaedter F. Bellec P. (2017). Resting-state network dysfunction in Alzheimer's disease: A systematic review and meta-analysis. Alzheimers Dement. 8, 73–85. 10.1016/j.dadm.2017.03.007 28560308 PMC5436069

[B8] Bai F. Liao W. Watson D. R. Shi Y. Yuan Y. Cohen A. D.. (2011). Mapping the altered patterns of cerebellar resting-state function in longitudinal amnestic mild cognitive impairment patients. J. Alzheimers. Dis. 23, 87–99. 10.3233/JAD-2010-101533 20930266

[B9] Bai F. Zhang Z. Watson D. R. Yu H. Shi Y. Yuan Y.. (2009). Abnormal functional connectivity of hippocampus during episodic memory retrieval processing network in amnestic mild cognitive impairment. Biol. Psychiatry. 65, 951–958. 10.1016/j.biopsych.2008.10.017 19028382

[B10] Battaglia F. P. Benchenane K. Sirota A. Pennartz C. M. Wiener S. I. (2011). The hippocampus: hub of brain network communication for memory. Trends Cogn. Sci. 15, 310–318. 10.1016/j.tics.2011.05.008 21696996

[B11] Biswal B. Y. F Haughton V. M. Hyde J. S. (1995). Functional connectivity in the motor cortex of resting human brain using echo-planar MRI. Magn. Reson. Med. 34, 537–541. 10.1002/mrm.1910340409 8524021

[B12] Boxer A. L. Kramer J. H. Du A. T. Schuff N. Weiner M. W. Miller B. L.. (2003). Focal right inferotemporal atrophy in AD with disproportionate visual constructive impairment. Neurology 61, 1485–1491. 10.1212/01.WNL.0000090568.34810.47 14663029 PMC2744649

[B13] Braak H. Braak E. (1985). On areas of transition between entorhinal allocortex and temporal isocortex in the human brain. normal morphology and lamina-specific pathology in Alzheimer's disease. Acta Neuropathol. 68, 325–332. 10.1007/BF00690836 4090943

[B14] Braak H. Braak E. (1991). Neuropathological stageing of Alzheimer-related changes. Acta Neuropathol. 82, 239–259. 10.1007/BF00308809 1759558

[B15] Chang C. C. Kramer J. H. Lin K. N. Chang W. N. Wang Y. L. Huang C. W.. (2010). Validating the Chinese version of the verbal learning test for screening Alzheimer's disease. J. Int. Neuropsychol. Soc. 16, 244–251. 10.1017/S1355617709991184 20003579 PMC3767760

[B16] Cheung R. W. Cheung M. C. Chan A. S. (2004). Confrontation naming in Chinese patients with left, right or bilateral brain damage. J. Int. Neuropsychol. Soc. 10, 46–53. 10.1017/S1355617704101069 14751006

[B17] Dickerson B. C. Brickhouse M. McGinnis S. Wolk D. A. (2017). Alzheimer's disease: The influence of age on clinical heterogeneity through the human brain connectome. Alzheimers Dement. 6, 122–135. 10.1016/j.dadm.2016.12.007 28239637 PMC5318292

[B18] Douaud G. Groves A. R. Tamnes C. K. Westlye L. T. Duff E. P. Engvig A.. (2014). A common brain network links development, aging, and vulnerability to disease. Proc. Natl. Acad. Sci. U.S.A. 111, 17648–17653. 10.1073/pnas.1410378111 25422429 PMC4267352

[B19] Eichenbaum H. (2017). Prefrontal-hippocampal interactions in episodic memory. Nat. Rev. Neurosci. 18, 547–558. 10.1038/nrn.2017.74 28655882

[B20] Folstein M. F. Folstein S. E. McHugh P. R. (1975). “Mini-mental state.” A practical method for grading the cognitive state of patients for the clinician. J. Psychiatr. Res. 12, 189–198. 10.1016/0022-3956(75)90026-6 1202204

[B21] Franzmeier N. Neitzel J. Rubinski A. Smith R. Strandberg O. Ossenkoppele R.. (2020). Functional brain architecture is associated with the rate of tau accumulation in Alzheimer's disease. Nat. Commun. 11:347. 10.1038/s41467-019-14159-1 31953405 PMC6969065

[B22] Henneman W. J. Sluimer J. D. Barnes J. van der Flier W. M. Sluimer I. C. Fox N. C.. (2009). Hippocampal atrophy rates in Alzheimer disease: added value over whole brain volume measures. Neurology 72, 999–1007. 10.1212/01.wnl.0000344568.09360.31 19289740 PMC2821835

[B23] Iba M. Guo J. L. McBride J. D. Zhang B. Trojanowski J. Q. Lee V. M. (2013). Synthetic tau fibrils mediate transmission of neurofibrillary tangles in a transgenic mouse model of Alzheimer's-like tauopathy. J. Neurosci. 33, 1024–1037. 10.1523/JNEUROSCI.2642-12.2013 23325240 PMC3575082

[B24] Jack C. R. Jr Knopman D. S. Jagust W. J. Shaw L. M. Aisen P. S. Weiner M. W.. (2010). Hypothetical model of dynamic biomarkers of the Alzheimer's pathological cascade. Lancet Neurol. 9, 119–128. 10.1016/S1474-4422(09)70299-6 20083042 PMC2819840

[B25] Jones D. T. Knopman D. S. Gunter J. L. Graff-Radford J. Vemuri P. Boeve B. F.. (2016). Cascading network failure across the Alzheimer's disease spectrum. Brain 139, 547–562. 10.1093/brain/awv338 26586695 PMC4805086

[B26] King M. Hernandez-Castillo C. R. Poldrack R. A. Ivry R. B. Diedrichsen J. (2019). Functional boundaries in the human cerebellum revealed by a multi-domain task battery. Nat. Neurosci. 22, 1371–1378. 10.1038/s41593-019-0436-x 31285616 PMC8312478

[B27] Kramer J. H. Jurik J. Sha S. J. Rankin K. P. Rosen H. J. Johnson J. K.. (2003). Distinctive neuropsychological patterns in frontotemporal dementia, semantic dementia, and Alzheimer disease. Cogn. Behav. Neurol. 16, 211–218. 10.1097/00146965-200312000-00002 14665820

[B28] Leech R. Sharp D. J. (2014). The role of the posterior cingulate cortex in cognition and disease. Brain 137(Pt 1), 12–32. 10.1093/brain/awt162 23869106 PMC3891440

[B29] Liu X. Chen X. Zheng W. Xia M. Han Y. Song H.. (2018). Altered functional connectivity of insular subregions in Alzheimer's disease. Front. Aging Neurosci. 10:107. 10.3389/fnagi.2018.00107 29695961 PMC5905235

[B30] Marek S. Siegel J. S. Gordon E. M. Raut R. V. Gratton C. Newbold D. J.. (2018). Spatial and temporal organization of the individual human cerebellum. Neuron 100, 977–993.e977. 10.1016/j.neuron.2018.10.010 30473014 PMC6351081

[B31] McKhann G. Drachman D. Folstein M. Katzman R. Price D. Stadlan E. M. (1984). Clinical diagnosis of Alzheimer's disease: report of the NINCDS-ADRDA work group under the auspices of department of health and human services task force on Alzheimer's disease. Neurology 34, 939–944. 10.1212/WNL.34.7.939 6610841

[B32] Mortamais M. Ash J. A. Harrison J. Kaye J. Kramer J. Randolph C.. (2017). Detecting cognitive changes in preclinical Alzheimer's disease: a review of its feasibility. Alzheimers. Dement. 13, 468–492. 10.1016/j.jalz.2016.06.2365 27702618

[B33] O'Callaghan C. Shine J. M. Hodges J. R. Andrews-Hanna J. R. Irish M. (2019). Hippocampal atrophy and intrinsic brain network dysfunction relate to alterations in mind wandering in neurodegeneration. Proc. Natl. Acad. Sci. U.S.A. 116, 3316–3321. 10.1073/pnas.1818523116 30718430 PMC6386688

[B34] Petersen R. C. Smith G. E. Waring S. C. Ivnik R. J. Tangalos E. G.. (1999). Mild cognitive impairment: clinical characterization and outcome. Arch. Neurol. 56, 303–308. 10.1001/archneur.56.3.303 10190820

[B35] Pievani M. Filippini N. van den Heuvel M. P. Cappa S. F. Frisoni G. B. (2014). Brain connectivity in neurodegenerative diseases–from phenotype to proteinopathy. Nat. Rev. Neurol. 10, 620–633. 10.1038/nrneurol.2014.178 25287597

[B36] Raichle M. E. (2015). The brain's default mode network. Annu. Rev. Neurosci. 38, 433–447. 10.1146/annurev-neuro-071013-014030 25938726

[B37] Raichle M. E. MacLeod A. M. Snyder A. Z. Powers W. J. Gusnard D. A. Shulman G. L. (2001). A default mode of brain function. Proc. Natl. Acad. Sci. U.S.A. 98, 676–682. 10.1073/pnas.98.2.676 11209064 PMC14647

[B38] Schröder J. Pantel J. (2016). Neuroimaging of hippocampal atrophy in early recognition of Alzheimer's disease–a critical appraisal after two decades of research. Psychiatry Res. Neuroimaging 247, 71–78. 10.1016/j.pscychresns.2015.08.014 26774855

[B39] Seeley W. W. Crawford R. K. Zhou J. Miller B. L. Greicius M. D. (2009). Neurodegenerative diseases target large-scale human brain networks. Neuron 62, 42–52. 10.1016/j.neuron.2009.03.024 19376066 PMC2691647

[B40] Spalletta G. Musicco M. Padovani A. Rozzini L. Perri R. Fadda L.. (2010). Neuropsychiatric symptoms and syndromes in a large cohort of newly diagnosed, untreated patients with Alzheimer disease. Am. J. Geriatr. Psychiatry 18, 1026–1035. 10.1097/JGP.0b013e3181d6b68d 20808086

[B41] Sridharan D. Levitin D. J. Menon V. (2008). A critical role for the right fronto-insular cortex in switching between central-executive and default-mode networks. Proc. Natl. Acad. Sci. U.S.A. 105, 12569–12574. 10.1073/pnas.0800005105 18723676 PMC2527952

[B42] Wang Z. Liang P. Jia X. Jin G. Song H. Han Y.. (2012). The baseline and longitudinal changes of PCC connectivity in mild cognitive impairment: a combined structure and resting-state fMRI study. PLoS ONE 7:e36838. 10.1371/journal.pone.0036838 22629335 PMC3356348

[B43] Xie C. Bai F. Yu H. Shi Y. Yuan Y. Chen G.. (2012). Abnormal insula functional network is associated with episodic memory decline in amnestic mild cognitive impairment. Neuroimage 63, 320–327. 10.1016/j.neuroimage.2012.06.062 22776459 PMC4513936

[B44] Zhang H. Y. Wang S. J. Liu B. Ma Z. L. Yang M. Zhang Z. J.. (2010). Resting brain connectivity: changes during the progress of Alzheimer disease. Radiology 256, 598–606. 10.1148/radiol.10091701 20656843

[B45] Zheng W. Liu X. Song H. Li K. Wang Z. (2017). Altered functional connectivity of cognitive-related cerebellar subregions in Alzheimer's disease. Front. Aging Neurosci. 9:143. 10.3389/fnagi.2017.00143 28559843 PMC5432635

[B46] Zhou Y. Dougherty J. H. Jr Hubner K. F. Bai B. Cannon R. L. Hutson R. K. (2008). Abnormal connectivity in the posterior cingulate and hippocampus in early Alzheimer's disease and mild cognitive impairment. Alzheimers Dement. 4, 265–270. 10.1016/j.jalz.2008.04.006 18631977

[B47] Zhu L. Shu H. Liu D. Guo Q. Wang Z. Zhang Z. (2018). Apolipoprotein E epsilon4 specifically modulates the hippocampus functional connectivity network in patients with amnestic mild cognitive impairment. Front. Aging Neurosci. 10:289. 10.3389/fnagi.2018.00289 30319395 PMC6170627

