# Supplementary material for: Posterior Cingulate Cortex Network Predicts Alzheimer's Disease Progression
Source: Front Aging Neurosci. 2020 Dec 15;12:608667. doi: 10.3389/fnagi.2020.608667 (PMC7770227; doi:10.3389/fnagi.2020.608667)
Supplement: Supplementary file 1 [file Table_1.DOCX]

**Supplementary Table 1.** Anatomical regions with significant gray matter atrophy rate changes between different groups

| **MNI Coordinates** | | | **Cluster size** | **Side** | **Anatomical Region** | **Maximum t-score** |
| --- | --- | --- | --- | --- | --- | --- |
| **x** | **y** | **z** |  |  |  |  |
| **HC < MCIs** | | |  |  |  |  |
| 30 | -77 | 21 | 826 | R | Precuneus | 4.28 |
| -39 | -83 | 20 | 3064 | L | Middle Occipital Gyrus | 4.15 |
| -36 | -15 | -32 | 2511 | L | Uncus | 3.83 |
| -18 | 36 | 42 | 1340 | L | Superior Frontal Gyrus | 3.78 |
| 56 | -56 | -15 | 1785 | R | Inferior Temporal Gyrus | 3.62 |
| **HC < MCIp** | | |  |  |  |  |
| 1 | -35 | 32 | 4656 | L | Cingulate Gyrus | 5.73 |
| 30 | -15 | -29 | 18364 | R | Parahippocampal Gyrus | 5.48 |
| -35 | 9 | -9 | 6531 | L | Insula | 4.76 |
| -17 | 18 | 60 | 2739 | L | Middle Frontal Gyrus | 3.96 |
| 33 | -42 | 41 | 544 | R | Sub-Gyral | 3.78 |
| **HC < AD** | | |  |  |  |  |
| -6 | -54 | 27 | 72645 | L | Posterior Cingulate | 5.88 |
| 36 | 15 | 56 | 5687 | R | Middle Frontal Gyrus | 4.75 |
| -19 | 37 | 45 | 5430 | L | Superior Frontal Gyrus | 3.99 |
| -32 | 2 | -14 | 545 | L | Extra-Nuclear | 3.65 |
| **MCIs < AD** | | |  |  |  |  |
| -12 | -48 | 38 | 4863 | L | Precuneus | 4.51 |
| 20 | -65 | -5 | 4158 | R | Lingual Gyrus | 4.21 |
| 39 | -69 | 47 | 1298 | R | Precuneus | 3.65 |
| **MCIp < AD** | | |  |  |  |  |
| 27 | -84 | 24 | 2406 | R | Cuneus | 3.85 |
| 12 | -69 | 54 | 747 | R | Precuneus | 3.11 |
| **MCIs < MCIp** | | |  |  |  |  |
| -2 | -32 | 30 | 1174 | L | Cingulate Gyrus | 4.74 |
| 26 | -8 | -27 | 1154 | R | Hippocampus | 3.78 |

Peak of group differences in GM atrophy rates with a threshold of FWE-corrected *p*-value < 0.05.

Abbreviations: AD, Alzheimer’s disease; HC, healthy controls; L, left; MCIp, mild cognitive impairment-progression; MCIs, mild cognitive impairment-stable; MNI, Montreal Neurological Institute; R, right.
