# Supplementary material for: Posterior Cingulate Cortex Network Predicts Alzheimer's Disease Progression
Source: Front Aging Neurosci. 2020 Dec 15;12:608667. doi: 10.3389/fnagi.2020.608667 (PMC7770227; doi:10.3389/fnagi.2020.608667)
Supplement: Supplementary file 2 [file Table_2.DOCX]

**Supplementary Table 2.** Brain areas involved in the synchronized degeneration networks of hippocampus and posterior cingulate cortex.

| **MNI Coordinates** | | | **Cluster  size** | **Side** | **Anatomical Region** | **Maximum t-score** |
| --- | --- | --- | --- | --- | --- | --- |
| **x** | **y** | **z** |  |  |  |  |
| **Hippocampus-SDN** | | | |  |  |  |
| 32 | -15 | -27 | 14175 | R | Parahippocampal Gyrus | 38.29 |
| 35 | -5 | -33 | - | R | Temporal Fusiform Cortex | 9.27 |
| 29 | 9 | -39 | - | R | Temporal Pole | 7.26 |
| -36 | -8 | -29 | 12978 | L | Temporal Fusiform Cortex | 8.49 |
| -29 | -24 | -17 | - | L | Parahippocampal Gyrus | 7.56 |
| -26 | 12 | -39 | - | L | Temporal Pole | 6.80 |
| -30 | 59 | -12 | 1666 | L | Frontal Pole | 5.89 |
| -44 | -59 | -32 | 3712 | L | Cerebellum | 5.81 |
| **PCC-SDN** | | | |  |  |  |
| -3 | -32 | 30 | 9210 | L | Cingulate Gyrus | 25.48 |
| -5 | -58 | 17 | - | L | Precuneous Cortex | 5.07 |
| -60 | -18 | 24 | 2549 | L | Postcentral Gyrus | 6.18 |
| 36 | -50 | -18 | 1323 | R | Temporal Occipital Fusiform Cortex | 5.10 |
| 54 | -33 | 3 | 1727 | R | Superior Temporal Gyrus | 4.53 |
| 48 | -5 | 15 | 1665 | R | Central Opercular Cortex | 4.27 |
| -8 | 18 | 33 | 1499 | L | Cingulate Gyrus | 4.18 |
| -2 | -57 | -27 | 955 | L | Cerebellum | 4.10 |
| -62 | -39 | -6 | 645 | L | Middle Temporal Gyrus | 4.08 |
| -32 | -38 | -24 | 599 | L | Temporal Fusiform Cortex, | 4.02 |
| -36 | -11 | 2 | 1751 | L | Insular Cortex | 3.97 |
| 11 | 15 | 50 | 517 | R | Superior Frontal Gyrus | 3.74 |
| 37 | -8 | -8 | 829 | R | Insular Cortex | 3.59 |

Abbreviations: L, left; MNI, Montreal Neurological Institute; PCC, posterior cingulate cortex; R, right; SDN, synchronized degeneration networks.
