# Supplementary material for: Posterior Cingulate Cortex Network Predicts Alzheimer's Disease Progression
Source: Front Aging Neurosci. 2020 Dec 15;12:608667. doi: 10.3389/fnagi.2020.608667 (PMC7770227; doi:10.3389/fnagi.2020.608667)
Supplement: Supplementary file 3 [file Data_Sheet_1.DOCX]

**Supplementary Figure 1.** Direct group comparison in annual gray matter atrophy rate between different study groups. Abbreviations: AD, Alzheimer’s disease; HC, healthy control; MCIp, mild cognitive impairment with progression to AD; MCIs, mild cognitive impairment stable without progression to AD.

**Supplementary Figure 2.** Network preference analysis with controlling SDN size. Left column: GOF scores of SDN with the fix network size from 1 to 10%, where green lines correspond to the hippocampus-SDN, and the blue lines to PCC-SDN. Right column: the spatial map of the hippocampus- and PCC-SDNs in the different network size.
