# Supplementary figures and images for: Posterior Cingulate Cortex Network Predicts Alzheimer's Disease Progression
Source: Front Aging Neurosci. 2020 Dec 15;12:608667. doi: 10.3389/fnagi.2020.608667 (PMC7770227; doi:10.3389/fnagi.2020.608667)

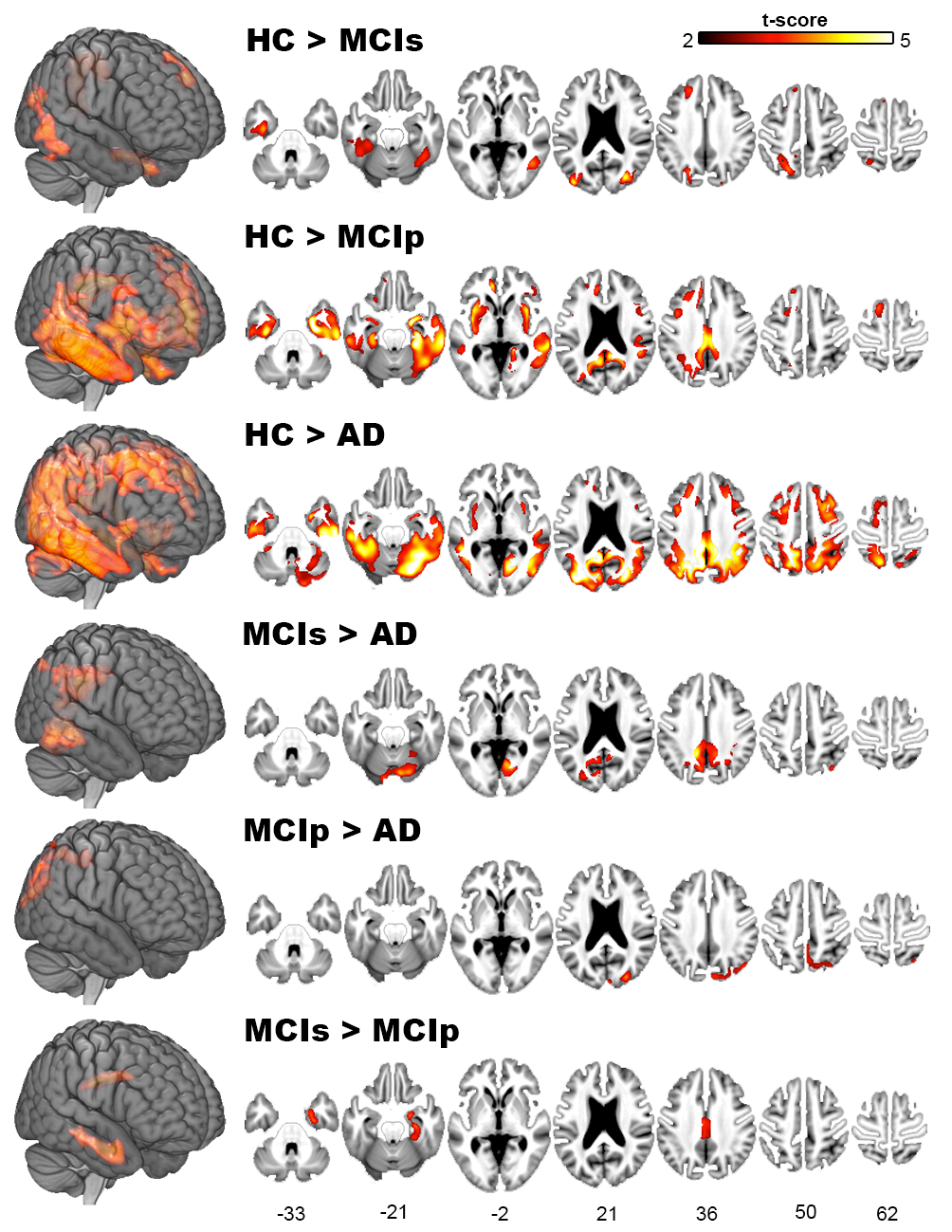

Supplement: Supplementary file 4 [file Image_1.TIF]

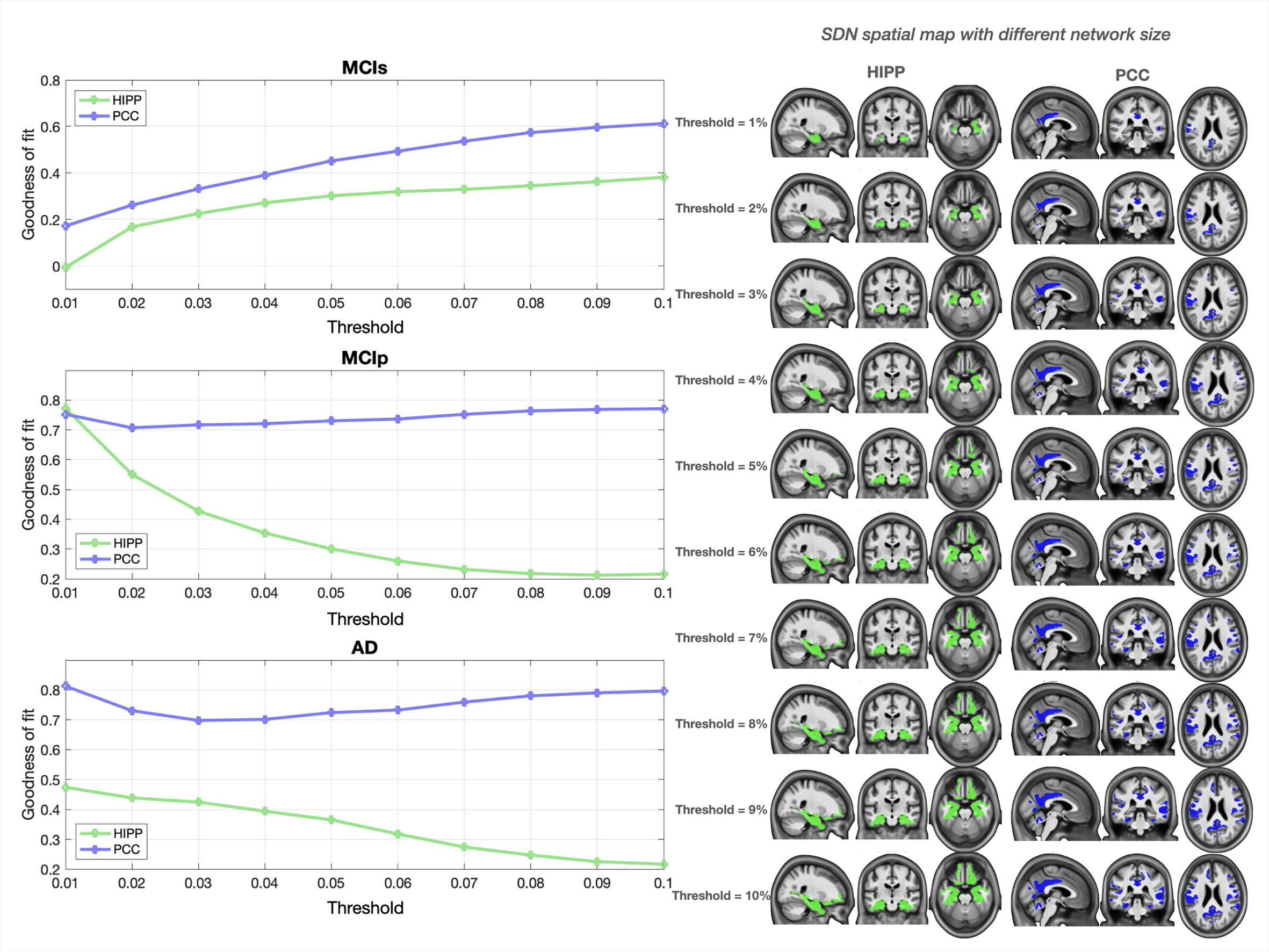

Supplement: Supplementary file 5 [file Image_2.TIF]
